# Redesigning Recombinase Specificity for Safe Harbor Sites in the Human Genome

**DOI:** 10.1371/journal.pone.0139123

**Published:** 2015-09-28

**Authors:** Mark C. Wallen, Thomas Gaj, Carlos F. Barbas

**Affiliations:** 1 The Skaggs Institute for Chemical Biology, The Scripps Research Institute, La Jolla, CA, 92037, United States of America; 2 Department of Chemistry, The Scripps Research Institute, La Jolla, CA, 92037, United States of America; 3 Department of Cell and Molecular Biology, The Scripps Research Institute, La Jolla, CA, 92037, United States of America; Imperial College London, UNITED KINGDOM

## Abstract

Site-specific recombinases (SSRs) are valuable tools for genetic engineering due to their ability to manipulate DNA in a highly specific manner. Engineered zinc-finger and TAL effector recombinases, in particular, are two classes of SSRs composed of custom-designed DNA-binding domains fused to a catalytic domain derived from the resolvase/invertase family of serine recombinases. While TAL effector and zinc-finger proteins can be assembled to recognize a wide range of possible DNA sequences, recombinase catalytic specificity has been constrained by inherent base requirements present within each enzyme. In order to further expand the targeted recombinase repertoire, we used a genetic screen to isolate enhanced mutants of the Bin and Tn21 recombinases that recognize target sites outside the scope of other engineered recombinases. We determined the specific base requirements for recombination by these enzymes and demonstrate their potential for genome engineering by selecting for variants capable of specifically recombining target sites present in the human CCR5 gene and the AAVS1 safe harbor locus. Taken together, these findings demonstrate that complementing functional characterization with protein engineering is a potentially powerful approach for generating recombinases with expanded targeting capabilities.

## Introduction

Genome engineering has emerged as a powerful approach for introducing custom alterations within biological systems [1]. Clinical applications of genome engineering, for instance, have the unique potential to treat the underlying causes of many diseases, ranging from monogenic disorders to the genetically complicated states associated with cancer. Recent advances in the field have focused on the development and application of site-specific nucleases. In particular, zinc-finger nucleases (ZFNs) [2–5], TAL effector nucleases (TALENs) [6–8] and CRISPR/Cas9 [9–12] have surfaced as tools capable of modifying both human cells and model organisms with high efficiency and flexibility. These enzymes induce targeted DNA double-strand breaks (DSBs), which stimulate the DNA damage response machinery and lead to the introduction of small insertions or deletions via non-homologous end joining (NHEJ) [13] or integration/correction by homology-directed repair (HDR) [3–5, 14]. However, despite their broad success, the utility of nuclease-based technologies is hampered by the formation of DSBs, which can be toxic to cells and lead to unknown and deleterious mutations at off-target sites [15–18]. Additionally, high rates of modification via HDR can be difficult to achieve in post-mitotic cell types. Together, these limitations underscore the need for the development of new technologies capable of inducing robust and safe genomic modifications.

Site-specific recombinases (SSRs; e.g., Cre and Flp) are a viable alternative to targeted nucleases for many applications of genome engineering [19]. SSRs are specialized enzymes that promote site-specific DNA rearrangements (i.e., integration, excision or inversion) between defined DNA segments [20]. SSRs cleave and re-ligate DNA autonomously and thus do not rely on the DNA repair machinery to introduce genomic modifications. However, because of their strict recognition capabilities, recombinase-mediated genome engineering has been limited to cells that contain either pre-introduced target sites or rare pseudo-recombination sites [21]. To overcome this, numerous protein engineering strategies have been developed to alter recombinase specificity [22]. Yet despite several successes [23, 24], these approaches have routinely led to enzymes with relaxed recognition specificities [25, 26], stemming from the fact that many recombinases display an intricate and overlapping network of catalytic and DNA-binding interactions.

**Fig 1:**
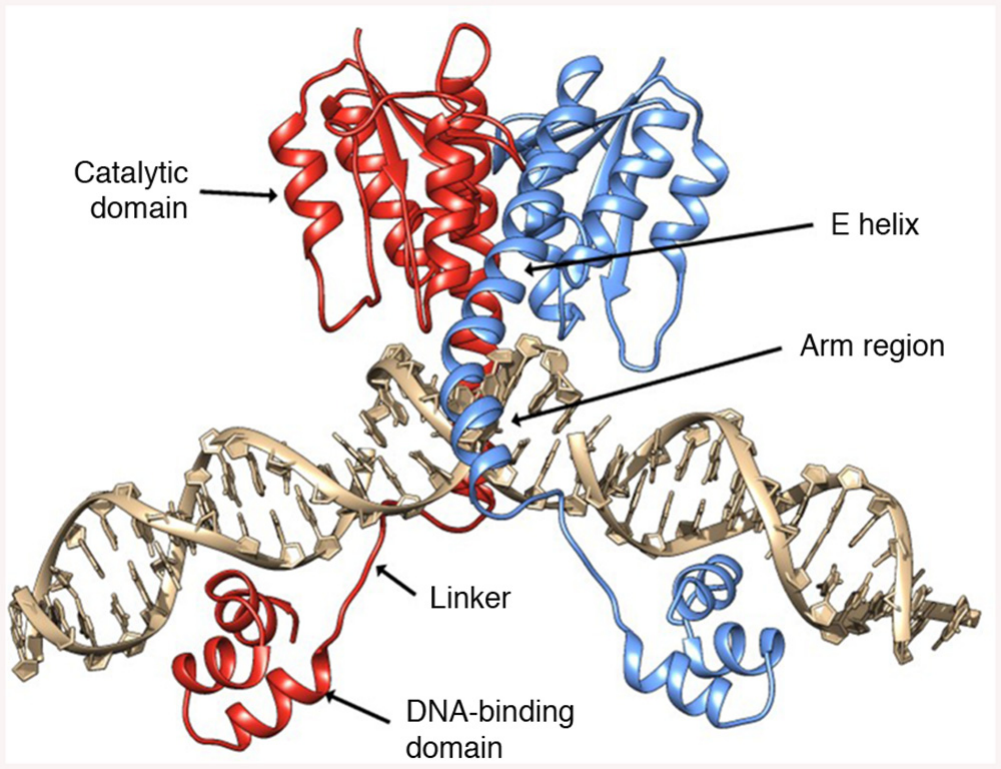
Serine recombinase structure. Important regions within each recombinase monomer (red and blue) are labeled. DNA shown in grey sticks. Native DNA-binding domains can be replaced with customizable zinc-finger or TAL effector domains to generate chimeric recombinases (PDB ID: 1GDT) [65].

In contrast to the SSRs described above, the resolvase/invertase family of serine recombinases [27] are modular in both structure and function, allowing the DNA-binding domains of these enzymes to be replaced without impairing catalytic function [28, 29] (Fig 1). Indeed, previous studies have shown that customizable Cys_2_-His_2_ zinc-finger [30–33] and TAL effector [34, 35] DNA-binding domains, which can be engineered to recognize a wide range of possible DNA sequences, can be fused to serine recombinase catalytic domains to generate synthetic enzymes with unique targeting capabilities [29, 36, 37]. In particular, zinc-finger recombinases (ZFRs) have shown the ability both to excise transgenic elements in a unidirectional manner [36] and to catalyze highly specific integration into the human genome [38]. We previously reported that substrate specificity profiling and selection of the recombinase DNA binding arm region could be used to generate a suite of catalytic domains with defined targeting capabilities that are capable of modifying user-defined target sites [39, 40]. While this approach was highly successful in creating recombinase variants with unique properties, conserved base constraints imposed by the recombinase catalytic domain prevented reprogramming toward all possible DNA sequences. However, as shown with the Sin and β recombinases [41], the use of catalytic domains with distinct base requirements offers an approach to circumvent those constraints and expand the suite of targetable sequences.

We thus set out to further expand the targeted recombinase repertoire by identifying catalytic domains compatible with our chimeric recombinase technology. We searched for enzymes that are homologous to prototypical serine recombinases, including β [42], Gin[43], Hin [44], Sin [45], Tn3 [46], and γδ [47], but exhibit distinct target site specificity. We hypothesized that such enzymes would be compatible with designed DNA-binding domains and amenable to specificity reprogramming. Our search led to the identification of two candidate enzymes, the Tn21 [48] and Bin [49] recombinases. However, in order to use these enzymes in the context of ZFRs, we set out to identify mutations that enable unrestricted recombination between minimal recombination sites.

Here we describe the generation of Bin and Tn21 recombinase variants that are capable of catalyzing unrestricted recombination between minimal crossover sites. We employed a genetic screen to determine the specific base requirements for these recombinases, and show that saturation mutagenesis and selection can be used to isolate unique variants capable of recombining target sites derived from the human CCR5 gene and the AAVS1 safe harbor locus. These results demonstrate that functional characterization and protein engineering can be used in tandem to generate recombinase variants with expanded targeting capabilities.

## Materials and Methods

### Plasmid construction

All ZFR target sites used in this study were introduced into the split gene reassembly plasmid (pBLA) as previously described [40, 50]. Briefly, GFPuv (Clontech), used as a stuffer fragment, was PCR amplified with the primers GFP-ZFR-XbaI-Fwd and GFP-ZFR-HindIII-Rev and digested with XbaI and HindIII. PCR products were ligated into the SpeI and HindIII restriction sites of pBLA to generate pBLA-ZFR substrates. All primer sequences are provided in Table A in S1 Document. Correct construction of each plasmid was verified by sequence analysis.

### Recombination assays

The genes encoding the Bin (UniProt ID: P19241) and Tn21 (UniProt ID: P04130) recombinase catalytic domains were synthesized (GeneArt) and fused to the H1 zinc-finger protein by overlap PCR (Table B in S1 Document), as previously described [51]. PCR products were digested with SacI and XbaI and ligated into the same restriction sites of pBLA. Ligations were transformed by electroporation into *E*. *coli* TOP10F′ (Life Technologies). After 1 hr recovery in Super Optimal Broth with Catabolite suppression (SOC) medium, cells were incubated with 5 mL of Super broth (SB) medium containing 30 μg/mL of chloramphenicol and cultured at 37°C with shaking (250 rpm). At 16 hr, cells were harvested by miniprep (Life Technologies) and 200 ng of pBLA plasmid was used to transform *E*. *coli* TOP10F’ cells. After 1 hr recovery in SOC, cells were plated on solid lysogeny broth (LB) medium with 30 μg/mL of chloramphenicol or 30 μg/mL of chloramphenicol and 100 μg/mL of carbenicillin, an ampicillin analogue. Recombination frequency was calculated as the number of colonies on chloramphenicol/carbenicillin plates divided by the number of colonies on chloramphenicol-only plates. Colony numbers were measured by automated colony counting using the GelDoc XR Imaging System (Bio-Rad).

### Selections

Bin and Tn21 catalytic domains were randomly mutagenized by error-prone PCR as described elsewhere [36, 52] and ligated into the SacI and XbaI sites of pBLA for selections. The BinQ arm region was mutagenized by overlap extension PCR as previously described [40]. Mutations were introduced into positions 122, 125, 129, 138 and 139 with the degenerate codon NNK (N: A, T, C or G; and K: G or T), which encodes all 20 amino acids. PCR products were digested with SacI and XbaI and ligated into the same restriction sites of pBLA. All library ligations were ethanol precipitated and used to transform *E*. *coli* TOP10F′. Library sizes were routinely measured to be ~5 x 10^6^. After 1 h recovery in SOC, cells were incubated in 100 mL of SB medium containing 30 μg/mL of chloramphenicol and cultured at 37°C with shaking. At 16 hr, cells were harvested and plasmid DNA was isolated by miniprep, followed by transformation of *E*. *coli* TOP10F′ with 3 μg of plasmid DNA. After 1 hr recovery in SOC, cells were incubated with 100 mL of SB medium containing 30 μg/mL of chloramphenicol and 100 μg/mL of carbenicillin and cultured at 37°C with shaking. At 16 hr, cells were harvested and plasmid DNA was purified by maxiprep (Life Technologies). Selected ZFRs were isolated by SacI and XbaI digestion and ligated into fresh vector for additional selection. Sequence analysis was performed on individual carbenicillin-resistant clones and recombination assays were performed on clones as described above.

### Specificity Profiling

GFPuv was PCR amplified using the primers GFP-mutant-ZFR-XbaI-Fwd, which contained randomized base substitutions at the 10–7, 6–4 or 3–2 base positions in the “left” 10-bp half-site of the ZFR target site, and GFP-ZFR-HindIII-Rev. PCR products were digested with XbaI and HindIII and ligated into SpeI and HindIII restriction sites of pBLA. Transformations were grown overnight for 16 hr in SB medium with 30 μg/mL chloramphenicol and harvested by miniprep to obtain a small library of substrates. BinQ and Tn21S were then cloned into pBLA substrate libraries and transformed as previously described. These cultures were allowed to grow in 30 μg/mL chloramphenicol for 4 hr before plating on solid LB medium with 30 μg/mL of chloramphenicol or 30 μg/mL of chloramphenicol and 100 μg/mL of carbenicillin. Chloramphenicol and carbenicillin resistant colonies were then sequenced for resolved ZFR target sites.

## Results

### Selection of active Bin and Tn21 catalytic domains

**Fig 2:**
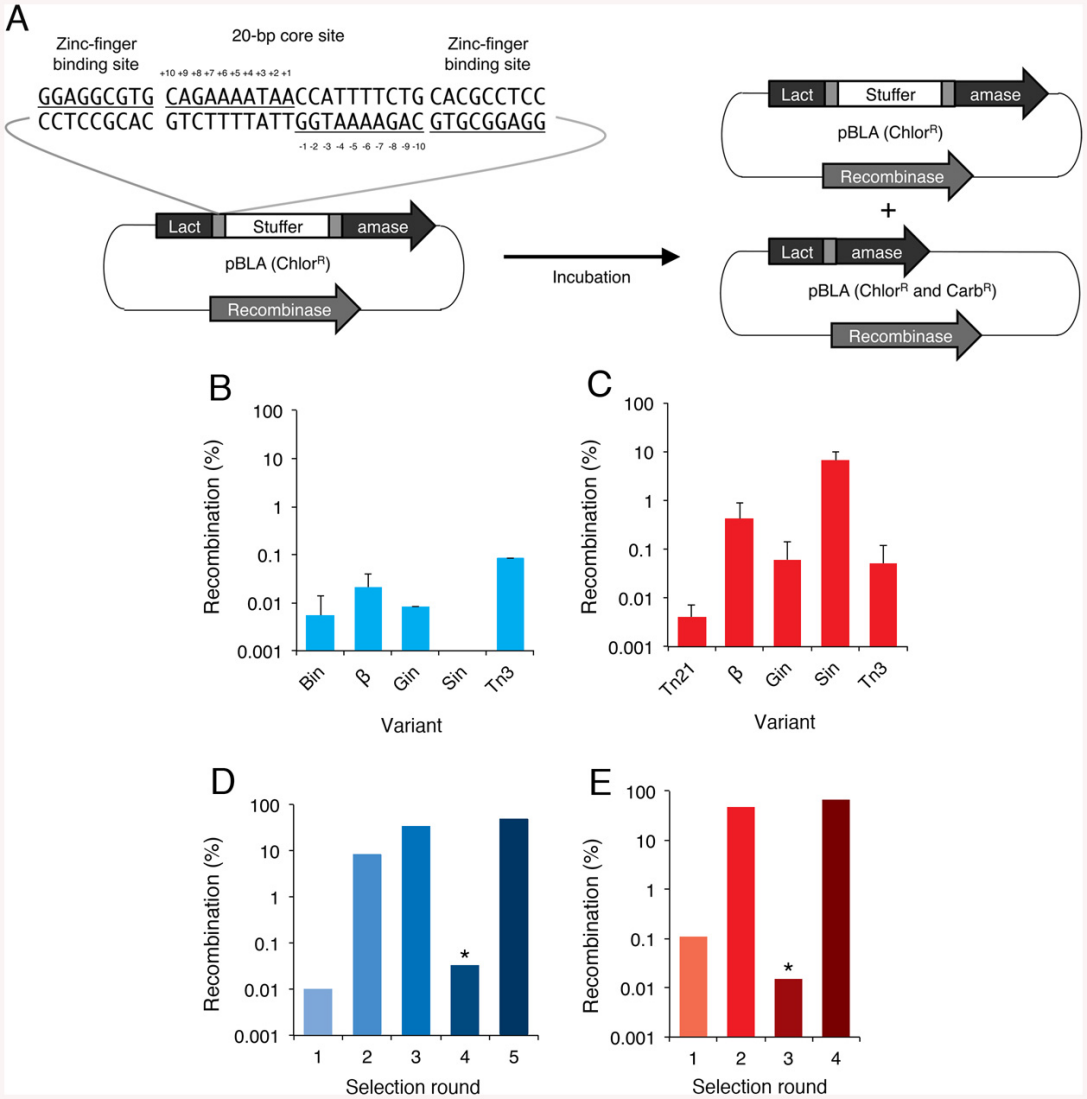
Selection of hyperactived Bin and Tn21 catalytic domains. **(A)** Schematic representation of the split gene reassembly system used to evaluate recombinase activity. Expression of an active recombinase leads to excision of the stuffer fragment (GFPuv), restoration of the β-lactamase reading frame and host cell resistance to carbenicillin (right, bottom). Full-length ZFR target site is shown and consists of a 20-bp core sequence recognized by the recombinase catalytic domain flanked by zinc-finger binding sites. Core positions are numbered. **(B, C)** Recombination activity of the native Bin, Tn21, and hyperactivated β, Gin, Sin and Tn3 catalytic domains in the context of ZFRs. Activity was measured on 20-bp core sites flanked by zinc-finger binding sites. Each core site was derived from the native **(B)** Bin and **(C)** Tn21 recombination sites (referred to as 20-Bin and 20-Tn21, respectively). Recombination was determined by split gene reassmbley as the percentage of recombined carbenicillin and chloremphenicol resistant clones versus total chloremphenicol resistant clones. Error bars indicate standard deviation (*n* = 3). **(D, E)** Selection of **(D)** Bin and **(E)** Tn21 variants that recombine 20-bp core sites derived from their native recombination sites, 20-Bin and 20-Tn21, respectively. Each recombinase catalytic domain was randomly mutated by error-prone PCR and analyzed for activity as a ZFR on a 20-bp core site-flanked by zinc-finger binding-sites. Asterisks indicate selection steps in which incubation time was deceased from 16 to 4 hr.

**Fig 3:**
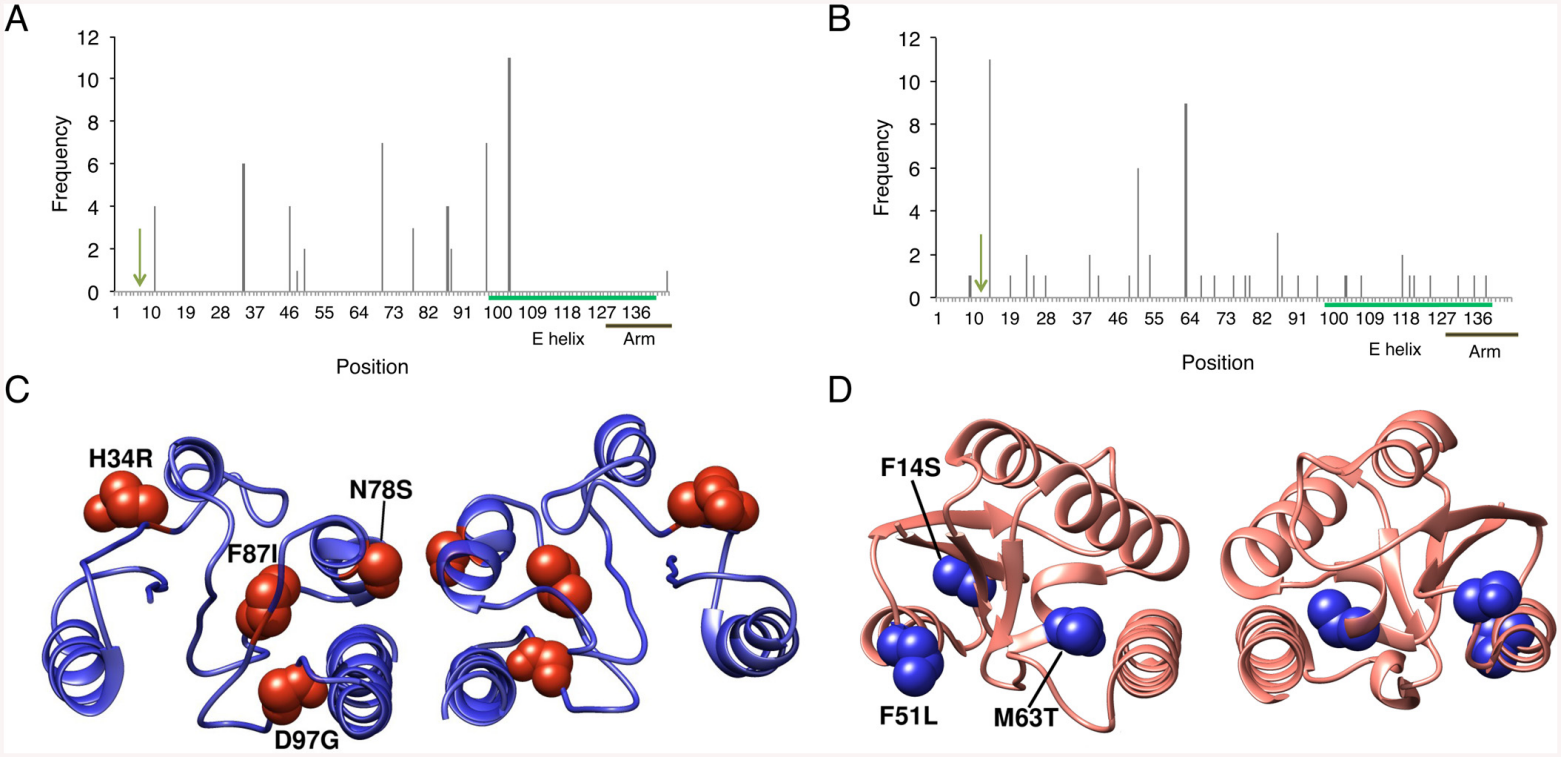
Analysis of hyperactivating mutations in the Bin and Tn21 catalytic domains. **(A, B)** Frequency and position of the mutations found to hyperactivatate the **(A)** Bin and **(B)** Tn21 catalytic domains. Green arrow indicates the catalytic serine residue **(C, D)** Crystal structures of **(C)** Gin-M114V (PDB ID: 3UJ3) [66] and **(D)** Sin-Q115R (PDB ID: 3PKZ) [67], which display homology to the Bin and Tn21 catalytic domains, respectively. Selected mutations present within **(C)** BinQ and **(D)** Tn21S are shown as red and blue spheres, respectively.

We began by analyzing the activity of the wild-type Bin and Tn21 catalytic domains on minimal crossover sites derived from their native recombination sites. These sites consist of a pseudo-symmetric 20-bp core sequence that contains two inverted 10-bp half-site regions. Specifically, we selected Bin and Tn21 for directed evolution due to their: (i) high sequence similarity to other serine recombinases, and (ii) unique core sites that address “gaps” within the targeted recombinase repertoire. Unlike Gin or any of its evolved variants, the recombination site recognized by Bin contains a TA base combination at positions 3–2, while the crossover site recognized by Tn21 includes G nucleotides at positions 6–4, a region typically restricted to A or T bases for other serine recombinases (Table 1). To measure activity, we used split gene reassembly, a method that directly links recombinase activity to antibiotic resistance in a bacterial host (Fig 2A) [41]. Both Bin and Tn21 demonstrated low levels of recombination (~0.1%) on their intended core sequences. Cross-comparative analysis revealed that hyperactivated variants of the Gin, Tn3, Sin and β catalytic domains also displayed negligible recombination on these substrates, while Sin showed ~10% recombination on the Tn21 core (Fig 2B and 2C). We next used antibiotic selection to identify mutations that enable unrestricted Bin- and Tn21-mediated recombination on their cognate core sequences. Similar approaches have been used to discover hyperactivating mutations for other serine recombinases, including Gin and Hin [53], Tn3 [54], γδ [55], Sin [41, 56] and β [41]. We used error-prone PCR to introduce ~2.5 and ~6 amino acid mutations into the Bin and Tn21 catalytic domains, respectively. We then fused each recombinase library to an unmodified copy of the H1 zinc-finger protein [57], which binds the sequence 5’-GGAGGCGTG-3’ and, in the split gene reassembly selection system, flanks the 20-bp core sequence recognized by the recombinase. After four and five rounds of selection with the Tn21 and Bin libraries, respectively, we observed a >1,000-fold increase in recombination via split gene reassembly (Fig 2C and 2D). We sequenced ~15 clones from each library and observed a number of recurrent mutations that were also commonly found together within singular clones. Among sequenced Bin variants, 65% contained the substitution G103D; 41% contained D97G and M70V/T; and 35% contained H34R (Fig 3A). For Tn21, 68% contained the mutation F14S; 56% contained M63T/V/I; 37% contained F51L/S; and 18% contained H86R/Y (Fig 3B). Hyperactivating mutations have previously been found to cluster near the recombinase E helix and have been proposed to either stabilize the active tetrameric configuration or destabilize the recombinase dimer. Surprisingly, only a few of the resulting Bin and Tn21 mutations were found to reside near the E helix (Fig 3C and 3D), with the majority of the mutations instead located near adjacent loops or the active site.

**Table 1:** Minimal core sites for select serine recombinases. Within the “left” 10-bp half-site, positions 6–4 are indicted by bold font and positions 3–2 are underlined.

Recombinase	Target Sequence	Abbreviation	Reference
β	CAAT **AGA**GT AT AC TTA TTTC	20B	[42]
Bin	CAGA **AAA**TA AC CA TTT TCTG	20-Bin	[49]
Gin α	CTGT **AAA**CC GA GG TTT TGGA	20G	[43]
Gin β	CTGT **AAA**GC GA GG TTT TGGA	-	[40]
Gin γ	CTGT **AAA**GT GA GG TTT TGGA	-	[40]
Gin δ	CTGT **AAA**CA GA GG TTT TGGA	-	[40]
Gin ζ	CTGT **AAA**TT GA GG TTT TGGA	-	[39]
Hin	TCAA **AAA**CC TT GG TTT TCAA	-	[44]
Sin	AATT **TGG**GT AC AC CCT AATC	20S	[45]
Tn21	GGTT **GAG**GC AT AC CCT AACC	20-Tn21	[48]
Tn3	CGAA **ATA**TT AT AA ATT ATCG	20T	[46]
γδ	CGAA **ATA**TT AT AA ATT ATCG	-	[47]

**Fig 4:**
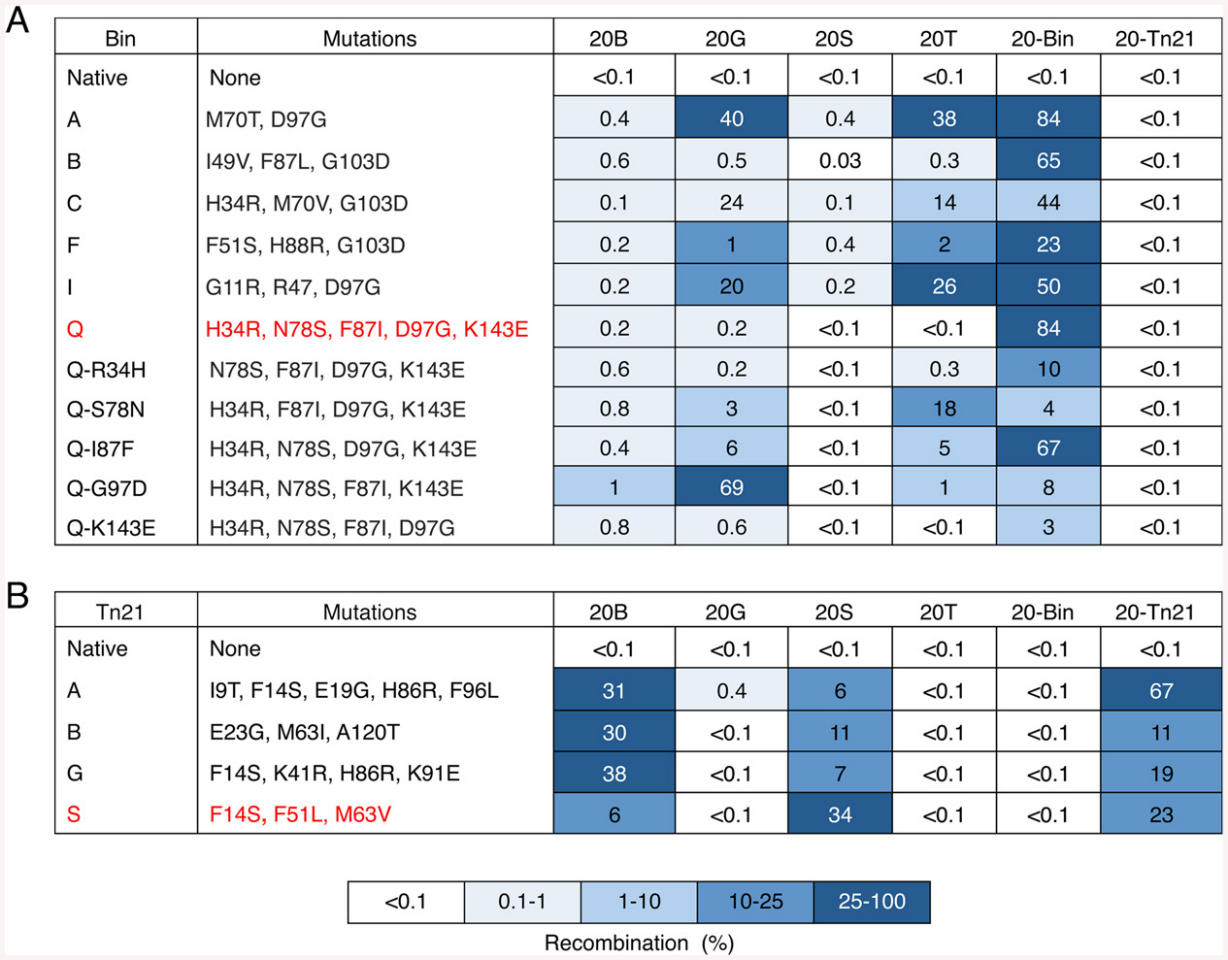
Recombination efficiency of selected Bin and Tn21 catalytic domain variants. The activity of selected **(A)** Bin and **(B)** Tn21 catalytic domains was evaluated against a panel of cognate and non-cognate target sites. Red highlighted variants were selected for further analysis. Recombination was determined by split gene assembly.

We next used split gene reassembly to measure the ability of individually selected Bin and Tn21 variants to recombine both cognate core sites (i.e., 20-Bin and 20-Tn21) and non-cognate (i.e., 20B, 20G, 20S and 20T) 20-bp core sites (Table 1). Among all analyzed Bin clones, BinQ (H34R, N78S, F87I, D97G and K143E) displayed the highest level of specificity for its intended DNA target (Fig 4A). In contrast to past studies, subtracting any single selected BinQ mutation dramatically reduced enzyme specificity and/or efficiency, indicating that the

BinQ mutations might work in concert to promote recombination (Fig 4A). Analysis of the selected Tn21 population revealed that all selected variants efficiently recombined their intended DNA targets (Fig 4B). Specifically, Tn21S (F14S, F51L and M63V) was selected for additional analysis because it efficiently recombined its cognate 20-bp core and it possesses the three most recurrent mutations identified within the selected Tn21 population. Interestingly, each evaluated Tn21 variant efficiently recombined the Sin and β core sites (Fig 4B), likely due to the presence of target site overlap and relaxed catalytic specificity.

### Specificity profiling of the BinQ catalytic domain

**Fig 5:**
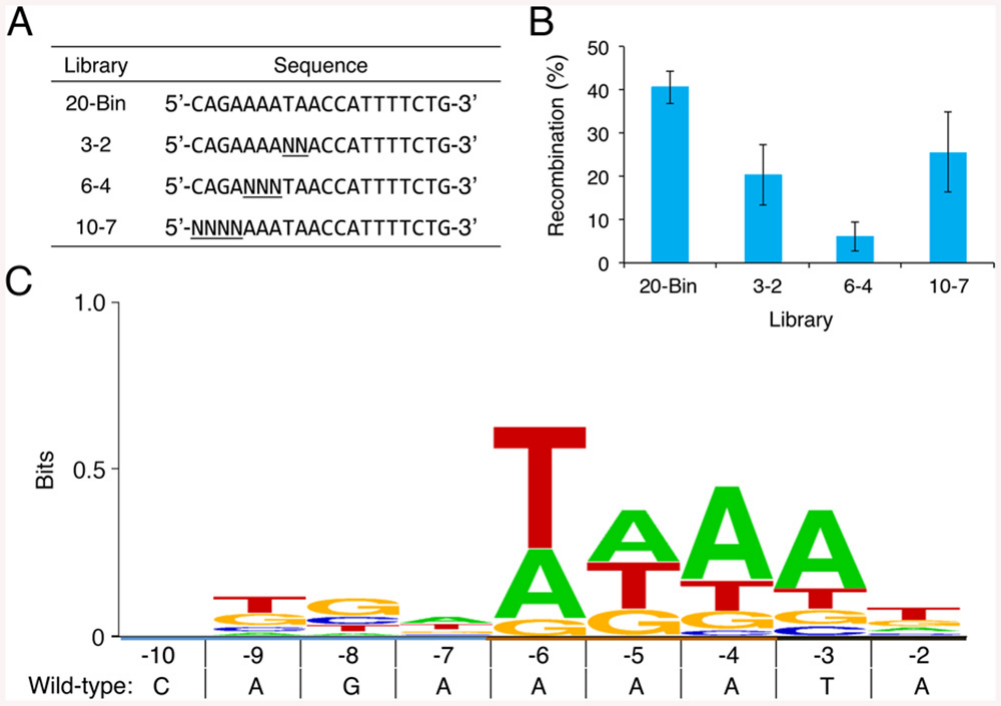
Specificity analysis of the BinQ catalytic domain. **(A)** Randomization strategy used for specificity profiling. Only “left” half-site bases were randomized. **(B)** Recombination by BinQ on each half-site library. “20-Bin” indicates the native 20-bp core site recognized by BinQ. Recombination was determined by split gene reassembly. Error bars indicate standard deviation (*n* = 3). Twenty clones were sequenced from each library output. **(C)** Weblogo of compiled data from all three substrate libraries, showing frequencies of bases tolerated at each position within the BinQ 10-bp half-site.

We next set out to develop a more detailed understanding of the determinants underlying BinQ and Tn21S target specificity. Based on previous reports utilizing split gene reassembly to identify the specific base requirements of the Sin and β recombinases at every position within a 10-bp half-site [41], we created Bin and Tn21 substrate libraries containing fully randomized base combinations within three regions: positions 10–7, 6–4, and 3–2 (Fig 5A). To ensure efficient recombination and sequencing of recombined products, mutations were introduced only within the “left” half-site of the recombinase target site. This approach facilitates straightforward retrieval of tolerated/recombined core sites by DNA sequencing, as the catalytic domain excises the two half-sites adjacent to the stuffer sequence during recombination. We began by evaluating the ability of BinQ to recombine DNA substrate libraries after 4 hr incubation in liquid culture, followed by antibiotic selection on LB agar plates. We sequenced the recombined substrates from ~20 individual transformants from each library in order to identify tolerated DNA substrates. Mutations in positions 6–4 were the most deleterious to activity, leading to a ~8-fold decrease in recombination, while substitutions within positions 10–7, and 3–2 reduced activity by less than 2-fold (Fig 5B). Sequence analysis revealed that BinQ possesses a specificity profile similar to the Gin recombinase, with no base determinants between positions 10–7 and a strong preference toward A or T at positions 6–4 (Fig 5C). However, unlike Gin or any of its evolved variants, BinQ demonstrated a bias for A or T bases at position 3 and T at position 2, indicating that its catalytic specificity can potentially fill gaps within the Gin targeting repertoire. Surprisingly, no consensus emerged for Tn21 (data not shown), suggesting that activation might have deleteriously broadened its catalytic specificity, as indicated by its off-target recombination on the Sin and β substrates (Fig 2C). Overall, these findings indicate that BinQ displays a distinct specificity profile that could complement existing recombinase for genomic targeting, and could be aided by more comprehensive studies in the future.

### Redesigning BinQ specificity for safe-harbor sites in the human genome

**Fig 6:**
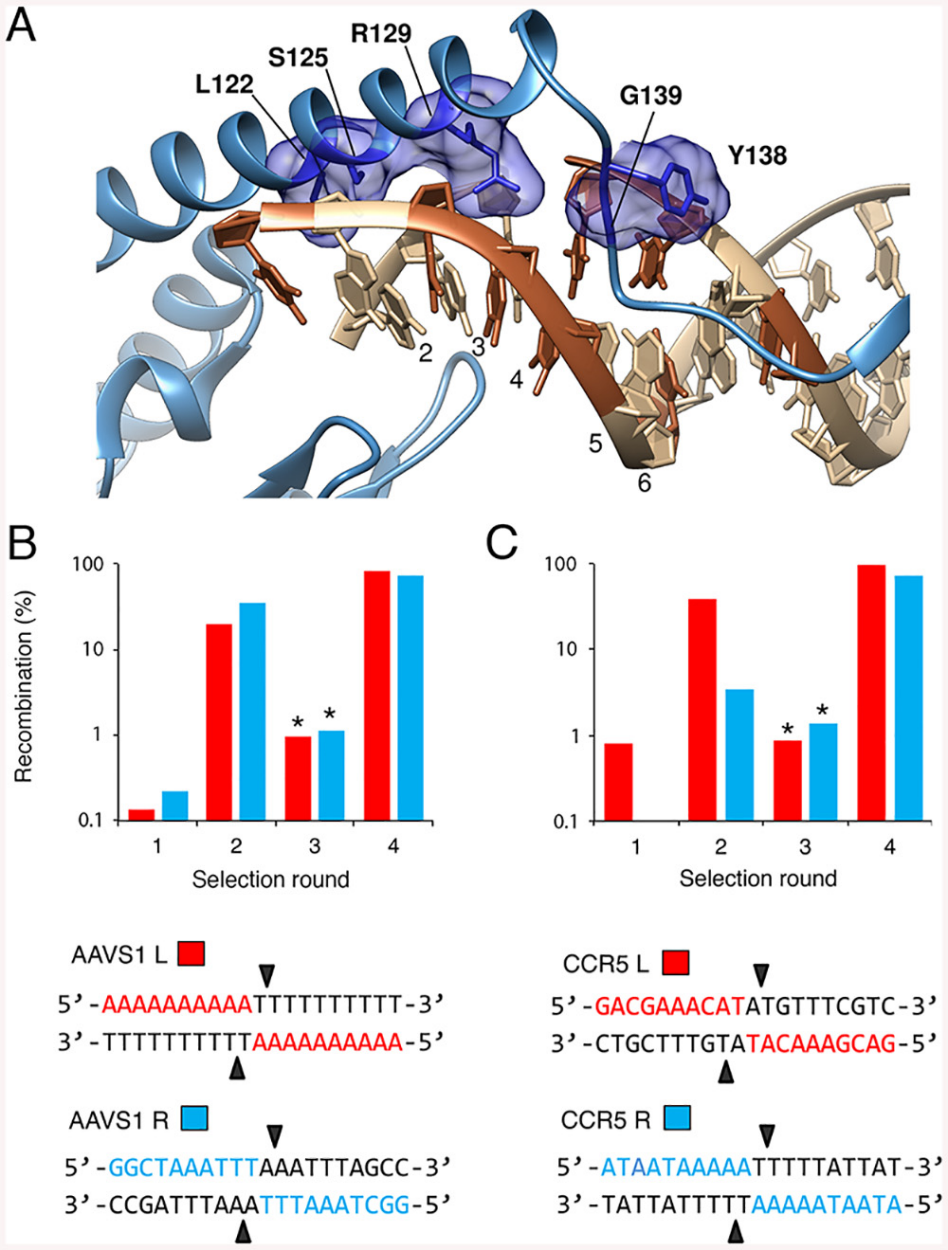
Redesigning BinQ catalytic specificity for the human CCR5 gene and AAVS1 safe harbor locus. **(A)** Structure of the γδ resolvase arm region (blue) in complex with DNA (PDB ID: 1ZR4) [65]. Residues selected for mutagenesis are shown as blue sticks and labeled with the corresponding residues in BinQ. Surrounding density is highlighted as space-filling blue. DNA positions within the 20-bp core half-site are indicated. (**B, C)** Selection of BinQ mutants that recombine the symmetrical versions of the “left” (blue) and “right” (red) **(B)** AAVS1 and **(C)** CCR5 target sites. Asterisks indicate selection steps in which incubation time was decreased from 16 to 4 hr. Sequences of the symmetrical AAVS1 L and R, and CCR5 L and R target sites are shown. Black arrows indicate DNA cleavage sites.

Within the human genome, there are several “safe harbor sites” that are capable of providing long-term gene expression in the absence of side effects [58], including the human chemokine (C-C motif) receptor 5 (CCR5) gene [59] and the AAVS1 locus (also known as the PPP1R12C locus) [60]. Because one potential application of engineered recombinases is site-specific integration of therapeutic factors into the human genome, we set out to re-engineer the specificity of the activated BinQ variant for both the human CCR5 and AAVS1 loci. We started by searching both the CCR5 and AAVS1 gene sequences for pseudo-recombination sites with: (i) similarity to the native BinQ target sites, particularly at positions 6–4, and (ii) potential flanking zinc-finger and TAL effector binding sites for eventual downstream studies. Using Zinc Finger Tools (http://scripps.edu/barbas/zfdesign/zfdesignhome.php) [61], we identified one possible target site within each locus composed primarily of high-scoring GNN and ANN triplets, with no predicted target site overlap (S1 Fig). Because Bin has a high sequence similarity to the Gin recombinase, we elected to modify the residues corresponding to those previously used to alter Gin specificity [40]. We constructed recombinase libraries by randomly mutagenizing five residues predicted to contact DNA at positions 3–2: Leu 122, Ser 125, Arg 129, Tyr 138 and Gly 139 (Fig 6A). Notably, these amino acid residues are located within the C-terminal arm region of the recombinase (Fig 1), which lies between the catalytic and DNA-binding domains, and mediates substrate recognition through direct interaction with the DNA.

**Fig 7:**
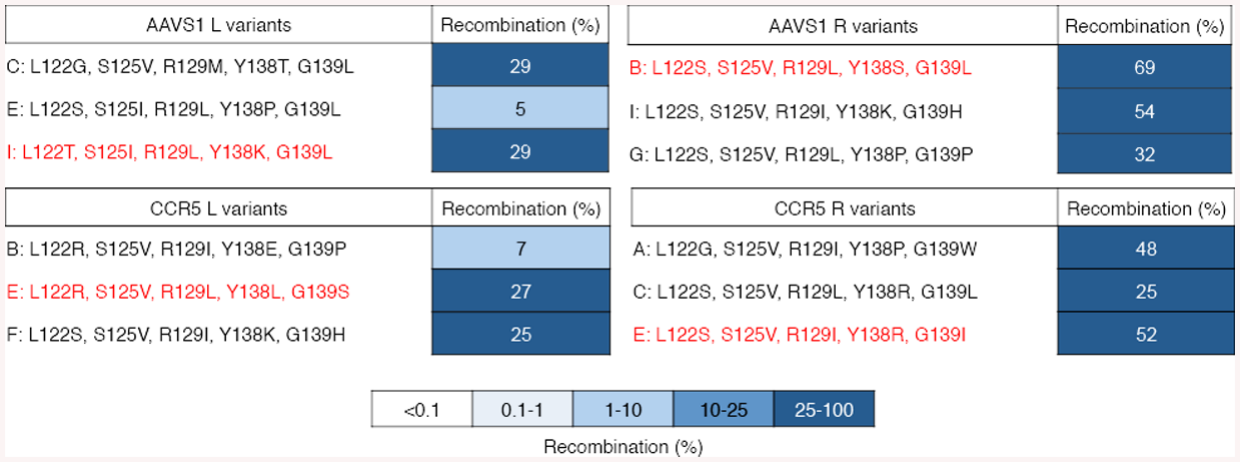
Recombination of the CCR5 and AAVS1 core sites by the selected BinQ variants. **(A)** Three BinQ mutants were evaluated for their ability to recombine the symmetrical AAVS1 L and R, and CCR5 L and R target sites that they were selected against. Selected mutations for each variant are shown. Red highlighted variants were selected for further analysis. Recombination was determined by split gene assembly.

Past studies have indicated that directed evolution on asymmetrical core sites promotes the selection of “generalist” recombinases with relaxed target specificity, as the enzyme must simultaneously recognize two dissimilar half-sites [36, 50, 62]. We thus hypothesized that when creating new recombinases for asymmetric sites, it might be necessary to generate a pair of “left” and “right” enzymes, each specific for half of the native genomic target, with the expectation that each individually evolved recombinase will function as a heterodimer with its partner in order to recombine the full-length target site. We therefore split the AAVS1 and CCR5 target sites at the dinucleotide core and created two asymmetrical, “left” and “right” DNA sequences for each genomic target, referred to as AAVS1 L, AAVS1 R, CCR5 L, and CCR5 R (Fig 6B and 6C). For selections, we fused the BinQ library to the H1 zinc-finger protein and cloned the subsequent ZFR library into substrate plasmids containing each recombinase target site, selecting for active variants by split gene reassembly. After four rounds of selection, we found that the activity of the BinQ population increased >1,000-fold on all DNA targets (Fig 6B and 6C). Sequencing revealed a high level of diversity for each library at BinQ positions 122 and 138, and strong conversion for hydrophobic residues at positions 125 and 129 (S2 Fig). Clonal analysis further revealed that the majority of selected recombinases displayed high (>25%) activity on their intended core sites (Fig 7). The most active variants are hereafter referred to as BinQ-AAVS1 L and R, and BinQ-CCR5 L and R (where “L” and “R” indicate the “left” and “right” symmetrical core sites the recombinase variant was evolved against, respectively).

**Fig 8:**
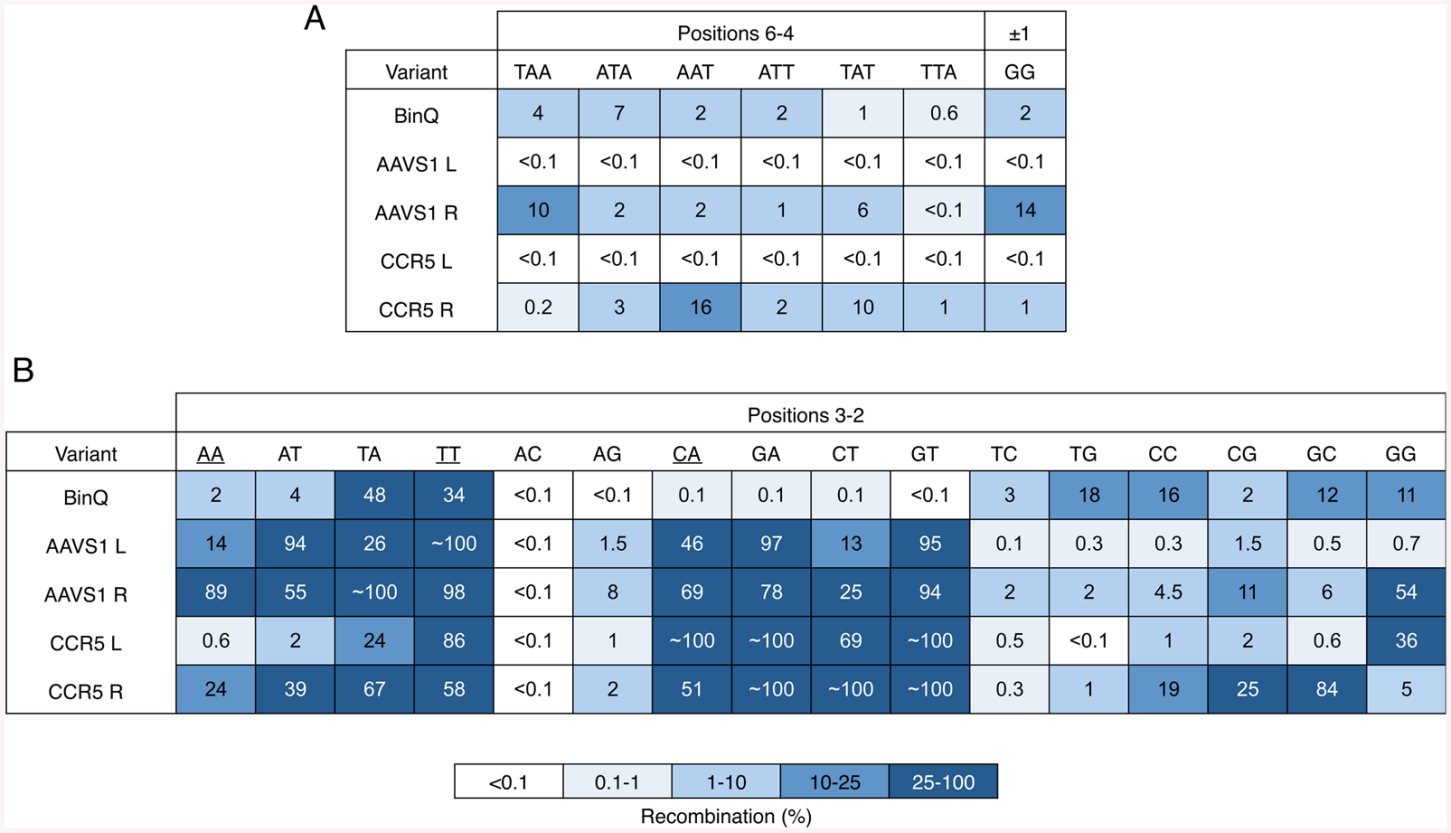
Specificity analysis of redesigned BinQ variants. Recombination by BinQ-CCR5 L and R, and BinQ-AAVS1 L and R on 20-Bin core sites containing **(A)** all posssible weak (W: A or T) substitutions within positions 6–4, or the dinucleotide core (±1) substitution GG and **(B)** all possible two-base combinations within positions 3–2. Recombination was determined by split gene assembly.

In order to more fully characterize the activity of each selected recombinase variant, we next evaluated the substrate specificity profile of BinQ-AAVS1 L and R, and BinQ-CCR5 L and R. This was achieved by introducing each possible weak base (A or T) substitution into positions 6–4, and each possible two-base combination into positions 3–2 within the 20-bp core site recognized by each BinQ variant. Compared to the parent clone, both BinQ-AAVS1 L and BinQ-CCR5 L displayed increased specificity for their intended target site, demonstrating low levels of recombination (<0.1%) on substrates containing even a single T substitution anywhere within positions 6–4 (Fig 8A). BinQ-AAVS1 L and BinQ-CCR5 L also exhibited minimal amounts of recombination when tested on core sites containing the dinucleotide core (±1) substitution GG. Similarly, both BinQ-AAVS1 R and BinQ-CCR5 R displayed a 10-fold decrease in recombination on substrates harboring any weak substitutions within positions 6–4 (Fig 8A). For positions 3–2, all evolved variants demonstrated some off-target activity, with substrates containing CA, GA, CT and GT substitutions yielding the highest levels of non-specific recombination (Fig 8B). Additionally, both BinQ-AAVS1 R and BinQ-CCR5 R showed increased off-target recombination for each substrate harboring a weak two-base substitution at positions 3–2. Together, these results demonstrate that enzyme variants capable of specific recombination of target sites from the CCR5 gene and AAVS1 locus can be generated by protein engineering methods.

## Discussion

In order for clinical and industrial applications of genome engineering to reach their full potential, improved methods capable of introducing targeted modifications in both a safe and efficient manner are needed. Most contemporary genome engineering processes rely on the use of targeted nucleases, such as ZFNs, TALENs and CRISPR/Cas9; however, these tools have the potential to introduce potentially toxic off-target DSBs and rely on the host cell machinery to facilitate targeted integration, a feature that could prevent their use in post-mitotic cells. SSRs, however, offer a potential solution to these problems, particularly for applications of therapeutic gene integration [63]. Yet despite their potential, new approaches for reconfiguring their specificity are needed.

Toward this goal, we incorporated two new recombinases, Bin and Tn21, into our chimeric recombinase repertoire. These enzymes show sequence similarity to prototypical serine resolvase/invertase family members but exhibit orthogonal target specificity, indicating their potential as tools capable of addressing gaps in the targeted recombinase sequence space. We used a positive antibiotic-based selection approach to isolate the hyperactivated variants BinQ and Tn21S, and showed that these mutants are capable of recombining minimal core sites on plasmid DNA with high efficiency in bacterial cells. To our knowledge, these are the first Bin and Tn21 variants shown to catalyze recombination between core sequences derived from their native recombination sites. Surprisingly, the majority of activating mutations selected in this study lie outside of the E helix, previously identified as a key region for altering enzyme stability and activity. This indicates that indirect effects between the selected mutations and the dimeric and tetrameric configurations may play a larger role in these recombinases compared to previously studied enzymes. Specifically, both BinQ and Tn21S contain substitutions at positions that encode large hydrophobic residues (F87I and F51L, respectively). In addition to these unique activating substitutions, we also identified an enhancing mutation within the Tn21 active site (F14S), suggesting that hyperactivation could be a product of an enhanced rate of catalysis. Future mutational studies could shed further light on the cooperative nature of the BinQ substitutions.

Site-specific integration of therapeutic factors into human safe harbor sites, such as CCR5 and AAVS1, could allow for long-term transgene expression without the risk of activating or inactivating other genes or regulatory elements. Despite previous advances made in expanding the targeted recombinase repertoire, conserved base requirements within the Gin, Tn3, Sin and β recombinase catalytic domains prevented their reprogramming for target sites present in such regions. Due to the finding that BinQ could recombine 20-bp core sites containing weak (WW) two-base combinations at positions 3–2, we hypothesized that it also could serve as an effective starting template for specificity reprogramming, as the only potential recombinase target sites within CCR5 and AAVS1 contained similar base compositions. This is in contrast to the Gin recombinase, which although amenable to protein engineering [38–40], has not yielded an evolved variant capable of recombining most WW base combinations at positions 3–2 within its 20-bp core. Compared to the parent enzyme, half of our BinQ variants selected for activity on the CCR5 and AAVS1 core sites showed improved specificity at positions 6–4 and the dinucleotide core. In contrast, all selected BinQ variants demonstrated reduced specificity at positions 3–2, indicating that: (i) more sophisticated mutagenesis strategies may be necessary to absolutely reprogram base specificity at these positions, and (ii) a complex interplay might exist between the targeted arm region residues and DNA. Future studies will be focused on using these catalytic domains with custom zinc-finger or TAL effector DNA-binding domains for site-specific integration into the endogenous CCR5 and AAVS1 target sites in human cells.

In conclusion, we show that specificity profiling in tandem with directed selection is an effective approach for generating recombinases with new properties with potential utility for genome engineering applications.

## Supporting Information

S1 Document (Table A) Primers used in this study. (Table B) Amino acid sequences of the proteins used in this study. (DOCX)

S1 Fig Potential ZFR target sites within the CCR5 gene and AAVS1 safe harbor locus. **(A)** AAVS1 and **(B)** CCR5 ZFR target sites selected for BinQ reprogramming. “TSO” indicates target site overlap that might arise from certain zinc-finger domains. Zinc-finger specificity, as determined by “base scoring”, was provided by the Zinc Finger Tools website.
(TIFF)

S2 Fig Amino acid mutation frequencies at positions targeted for randomization in the BinQ catalytic domain. >20 variants were sequenced from each library after four rounds of selection.
(TIFF)
