# Supplementary material for: Redesigning Recombinase Specificity for Safe Harbor Sites in the Human Genome
Source: PLoS One. 2015 Sep 28;10(9):e0139123. doi: 10.1371/journal.pone.0139123 (PMC4587366; doi:10.1371/journal.pone.0139123)
Supplement: S1 Document — (DOCX) [file pone.0139123.s001.docx]

**Supplementary Data**

**Redesigning Recombinase Specificity for Safe Harbor Sites in the Human Genome**

Mark C. Wallen^1, 2, 3^, Thomas Gaj^1, 2, 3, †^ and Carlos F. Barbas III^1, 2, 3, ‡^

^1^The Skaggs Institute for Chemical Biology, The Scripps Research Institute, La Jolla, CA, 92037, USA.

^2^Department of Chemistry, The Scripps Research Institute, La Jolla, CA, 92037, USA.

^3^Department of Cell and Molecular Biology, The Scripps Research Institute, La Jolla, CA, 92037, USA.

^†^Present address: Department of Chemical and Biomolecular Engineering, University of California, Berkeley, CA, 94720, USA.

^‡^Deceased

Correspondence to Mark C. Wallen: Department of Cell and Molecular Biology, The Scripps Research Institute, La Jolla, CA 92037, USA. Email: mcwallen@scripps.edu

**Short title:** Redesigning recombinase specificity

>GFP-ZFR-Bin-XbaI Fwd

TTAATTAAGAGTCTAGACGGAGGCGTGCAGAAAATAACCATTTTCTGCACGCCTCCAGATCTAGGAGGAATTTAAAATGAG

>GFP-ZFR-HindIII Rev

GATGAACTGTACAAATAACTGCAGGGAGGCGTGCAGAAAATAACCATTTTCTGCACGCCTCCCCAAGCTTCTCTAGGTCAGT

>GFP-ZFR-Bin(3,2)-XbaI Fwd

TTAATTAAGAGTCTAGACGGAGGCGTGCAGAAAANNACCATTTTCTGCACGCCTCCAGATCTAGGAGGAATTTAAAATGAG

>GFP-ZFR-Bin(6-4)-XbaI Fwd

TTAATTAAGAGTCTAGACGGAGGCGTGCAGANNNTAACCATTTTCTGCACGCCTCCAGATCTAGGAGGAATTTAAAATGAG

>GFP-ZFR-Bin(10-7)-XbaI Fwd

TTAATTAAGAGTCTAGACGGAGGCGTGNNNNAAATAACCATTTTCTGCACGCCTCCAGATCTAGGAGGAATTTAAAATGAG

>GFP-ZFR-Tn21-XbaI Fwd

TTAATTAAGAGTCTAGAAGGAGGCGTGGGTTGAGGCATACCCTAACCCACGCCTCCAGATCTAGGAGGAATTTAAAATGAG

>GFP-ZFR-Tn21-HindIII Rev

ACTGACCTAGAGAAGCTTTTGGAGGCGTGGGTTAGGGTATGCCTCAACCCACGCCTCCCTGCAGTTATTTGTACAGTTCATC

> GFP-ZFR-Tn21(3,2)-XbaI Fwd

TTAATTAAGAGTCTAGAAGGAGGCGTGGGTTGAGNNATACCCTAACCCACGCCTCCAGATCTAGGAGGAATTTAAAATGAG

> GFP-ZFR-Tn21(6-4)-XbaI Fwd

TTAATTAAGAGTCTAGAAGGAGGCGTGGGTTNNNGCATACCCTAACCCACGCCTCCAGATCTAGGAGGAATTTAAAATGAG

> GFP-ZFR-Tn21(10-7)-XbaI Fwd

TTAATTAAGAGTCTAGAAGGAGGCGTGNNNNGAGGCATACCCTAACCCACGCCTCCAGATCTAGGAGGAATTTAAAATGAG

**Table A. Primers Used in this Study.**

>TN21

MTGQRIGYIRVSTFDQNPERQLEGVKVDRAFSDKASGKDVKRPQLEALISFARTGDTVVVHSMDRLARNLDDLRRIVQTLTQRGVHIEFVKEHLSFTGEDSPMANLMLSVMGAFAEFERALIRERQREGIALAKQRGAYRGRKK

>Bin

MKIGYARVSTGLQNLNLQEDRLNQYGCEKIFSDHISGAKSKRPGLDRAIEFARSGDTIVVWRLDRLGRNMADLITLVNELNNRGVSFHSLEENITMDKSSSTGQLLFHLFAAFAEFERNLILERSSAGRIAARARGRYGGRPEK

>H1 zinc-finger

sGSGEPYKCPECGKSFSRSDVLVRHQRTHTGEKPYKCPECGKSFSDPGHLVRHQRTHTGEKPYKCPECGKSFSQSSHLVRHQRTHTGKKT

>Gin (H106Y)

MLIGYVRVSTNDQNTDLQRNALVCAGCEQIFEDKLSGTRTDRPGLKRALKRLQKGDTLVVWKLDRLGRSMKHLISLVGELRERGINFRSLTDSIDTSSPMGRFFFYVMGALAEMERELIIERTMAGLAAARNKGRIGGRPPK

>TN3 (G70S, D102Y, E124Q)

MRIFGYARVSTSQQSLDIQIRALKDAGVKANRIFTDKASGSSTDREGLDLLRMKVEEGDVILVKKLDRLSRDTADMIQLIKEFDAQGVAVRFIDDGISTDGYMGQMVVTILSAVAQAERRRILQRTNEGRQEAKLKGIKFGRRRT

>Sin (Q115R)

MIIGYARVSSIDQNLERQLDNLKTFGVEKIFTEKQSGKSVENRPVFQEALNFVRMGDRFVVESIDRLGRNYDEIIETVNYLKEKDVQLMITSLPMMNEVTGNPLLDKFMKDLIIQILAMVSEQERNESKRRQAQGIQVAKEKGVYKGRPLL

>Beta (N95D)

MAKIGYARVSSKEQNLDRQLQALQGVSKVFSDKLSGQSVERPQLQAMLNYIREGDIVVVTELDRLGRNNKELTELMNAIQQKGATLEVLNLPSMDGIEDENLRRLINNLVIELYKYQAESERKRIKERQAQGIEIAKSKGKFKGRQHK

**Table B. Amino Acid Sequences of Proteins used in this Study.**
