# Supplementary figures and images for: Redesigning Recombinase Specificity for Safe Harbor Sites in the Human Genome
Source: PLoS One. 2015 Sep 28;10(9):e0139123. doi: 10.1371/journal.pone.0139123 (PMC4587366; doi:10.1371/journal.pone.0139123)

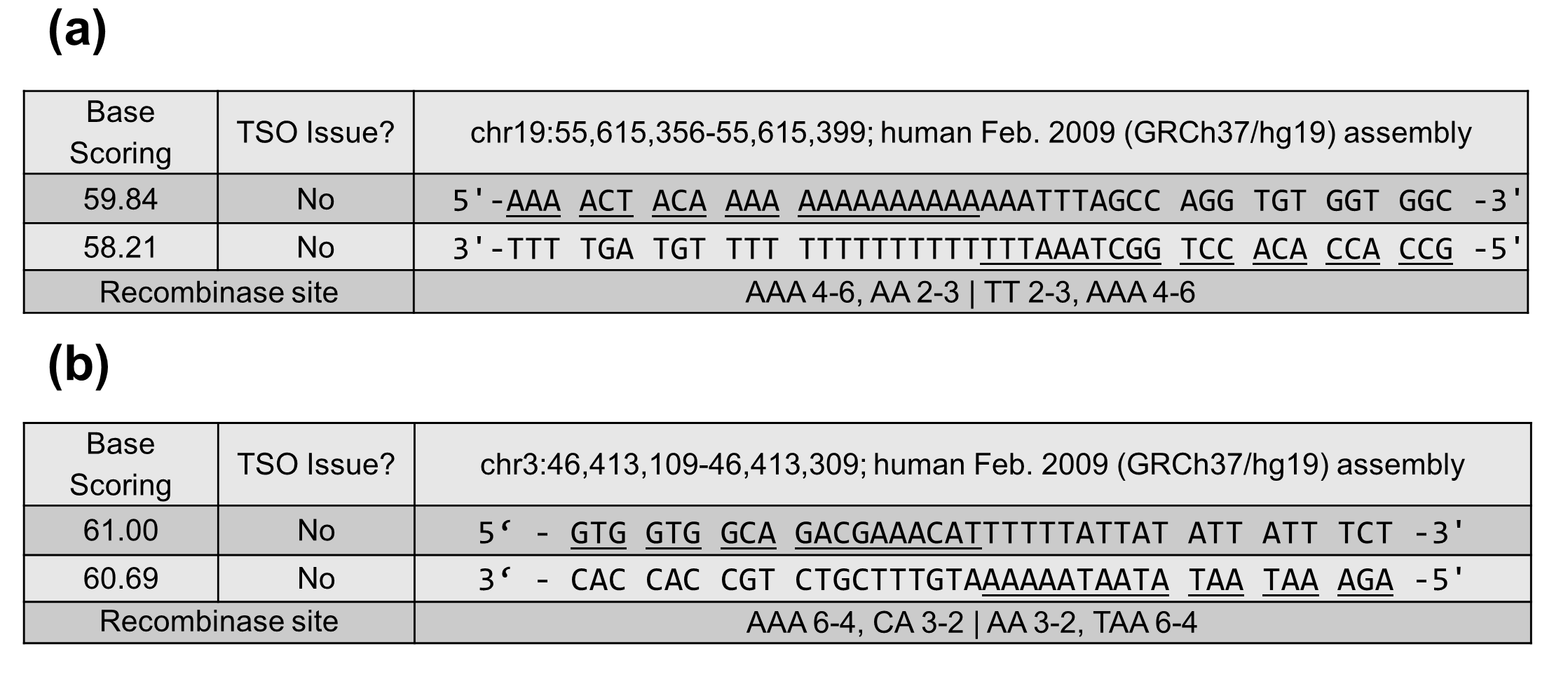

Supplement: S1 Fig — (A) AAVS1 and (B) CCR5 ZFR target sites selected for BinQ reprogramming. “TSO” indicates target site overlap that might arise from certain zinc-finger domains. Zinc-finger specificity, as determined by “base scoring”, was provided by the Zinc Finger Tools website. (TIFF) [file pone.0139123.s002.tiff]

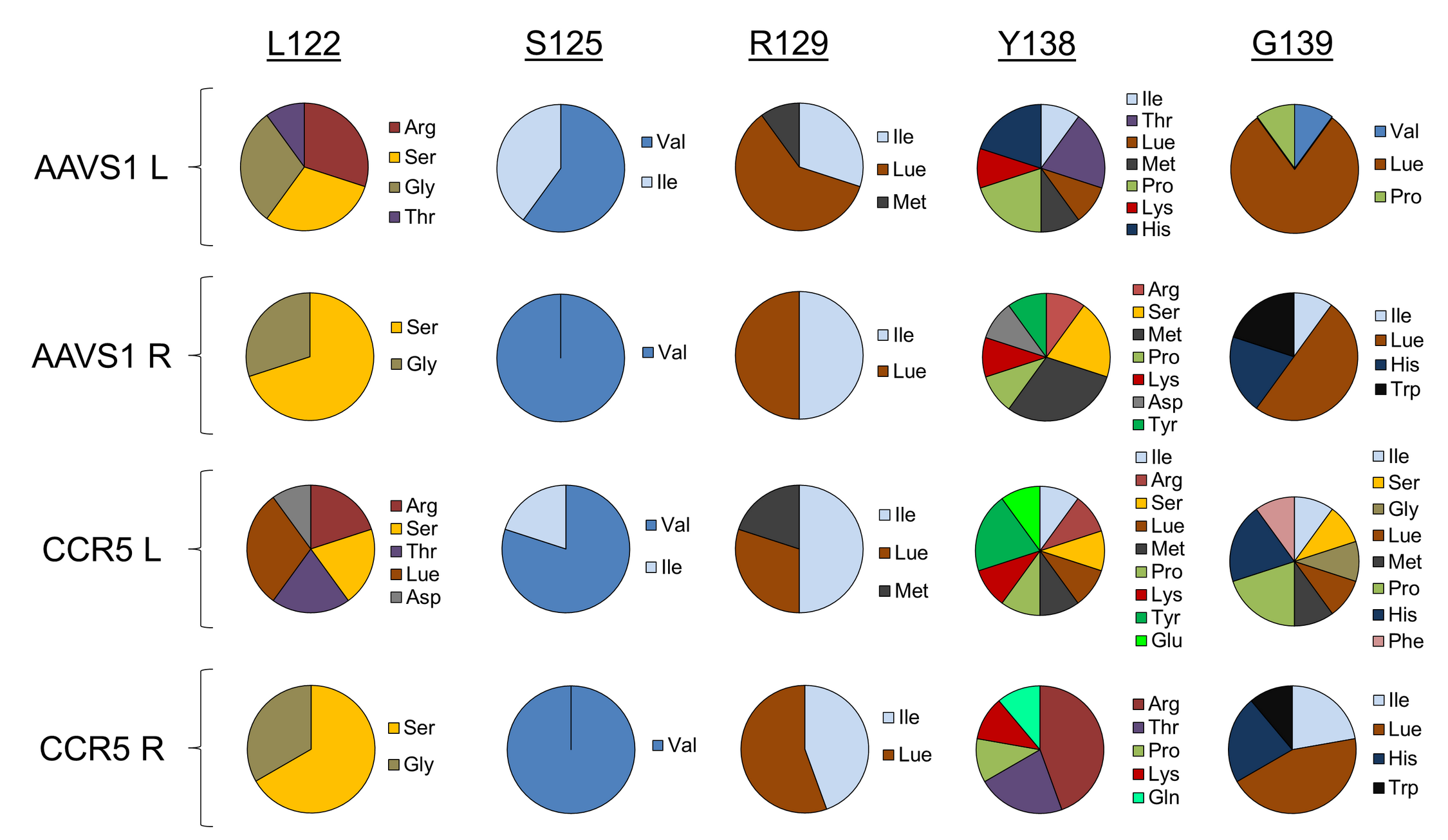

Supplement: S2 Fig — >20 variants were sequenced from each library after four rounds of selection. (TIFF) [file pone.0139123.s003.tiff]
